# Emerging Roles of Lipophagy in Cancer Metastasis

**DOI:** 10.3390/cancers14184526

**Published:** 2022-09-19

**Authors:** Haimeng Yin, Ying Shan, Tian Xia, Yan Ji, Ling Yuan, Yiwen You, Bo You

**Affiliations:** 1 Institute of Otolaryngology Head and Neck Surgery, Affiliated Hospital of Nantong University, Xisi Road 20, Nantong 226001, China; 2 Medical School, Nantong University, Qixiu Road 19, Nantong 226001, China; 3 Department of Otorhinolaryngology Head and Neck surgery, Affiliated Hospital of Nantong University, Xisi Road 20, Nantong 226001, China

**Keywords:** lipophagy, lipid metabolism, cancer metastasis

## Abstract

**Simple Summary:**

Metastasis is the main cause of death in patients with malignant tumors worldwide. Mounting evidence suggests lipid droplet metabolism is involved in the process of metastasis. As a mechanism to selectively degrade lipid droplets, the current research on lipophagy and tumor metastasis is quite limited. This review summarizes the crosstalk among lipophagy, tumor lipid metabolism and cancer metastasis, which will provide a new reference for the development of effective targeted drugs.

**Abstract:**

Obesity is a prominent risk factor for certain types of tumor progression. Adipocytes within tumor stroma contribute to reshaping tumor microenvironment (TME) and the metabolism and metastasis of tumors through the production of cytokines and adipokines. However, the crosstalk between adipocytes and tumor cells remains a major gap in this field. Known as a subtype of selective autophagy, lipophagy is thought to contribute to lipid metabolism by breaking down intracellular lipid droplets (LDs) and generating free fatty acids (FAs). The metastatic potential of cancer cells closely correlates with the lipid degradation mechanisms, which are required for energy generation, signal transduction, and biosynthesis of membranes. Here, we discuss the recent advance in the understanding of lipophagy with tumor lipid metabolism and review current studies on the roles of lipoghagy in the metastasis of certain human malignancies. Additionally, the novel candidate drugs targeting lipophagy are integrated for effective treatment strategies.

## 1. Introduction

Cancer metastasis is the progression of cancer cells escaping from a primary site to distant organs that they gradually colonize [1]. Cancer metastasis is a dynamic process with multi-factor participation and multi-stage development involving tumor cells themselves, the interaction between the tumor and the microenvironment, etc. Given its systemic nature and the resistance of disseminated tumor cells to existing therapies, metastatic disease accounts for 90% malignant cancer-related deaths [2]. Thus, our capacity to interdict the process of metastasis largely determines the therapeutic efficacy. Based on the clinical realities, attention to implicating specific molecules in biological aspects of the metastasis has increased [3].

Lipophagy is a source of alternative energy in times of nutrient scarcity [4]. In a broad sense, lipophagy include two types: chaperone-mediated lipophagy and macrolipophagy; in a narrow sense, macrolipophagy is identified as lipophagy because it occurs more frequently [5]. During the selective autophagic degradation of lipid droplets (LDs), the phosphorylation of perilipins is firstly triggered by protein kinase A [6]. LD surface proteins PLIN2 and PLIN3 are next recognized by Hsc70 and bind to lysosomal membrane receptor LAMP2, contributing to a degradation progress called chaperone-mediated lipophagy [7]. Then, the LDs are exposed for degradation in two ways: (1) lipases represented by lipase degrade LDs directly into fatty acids (FAs) by lipolysis [8]; (2) Rab7 and Rab10 recruit lysosome and LC3-positive autophagic membranes to LDs, respectively [9,10], leading to a progress termed macrolipophagy. Lipophagy was firstly described in liver cells [4], since then, the function of lipophagy in adipocytes [11], neurons [12], renal tubular cells [13], prostate carcinoma cells [14], breast cancer cells [15] and foam macrophages [16] have been discovered gradually. Remarkably, an effective way to monitor the lipophagy process by a pH-sensitive spirocyclization strategy makes it easier to visualize the closely related organelles [17].

Notably, recent studies indicate that lipophagy not only supports fuels for cancer metastasis [15], but also participates in each step of metastasis [18,19]. So, understanding the latest potential roles of lipophagy in common malignancies to find new therapeutic targets is of great importance.

## 2. Overview of Lipophagy in Tumor Lipid Metabolism

### 2.1. Steps of Lipophagy

**Figure 1:**
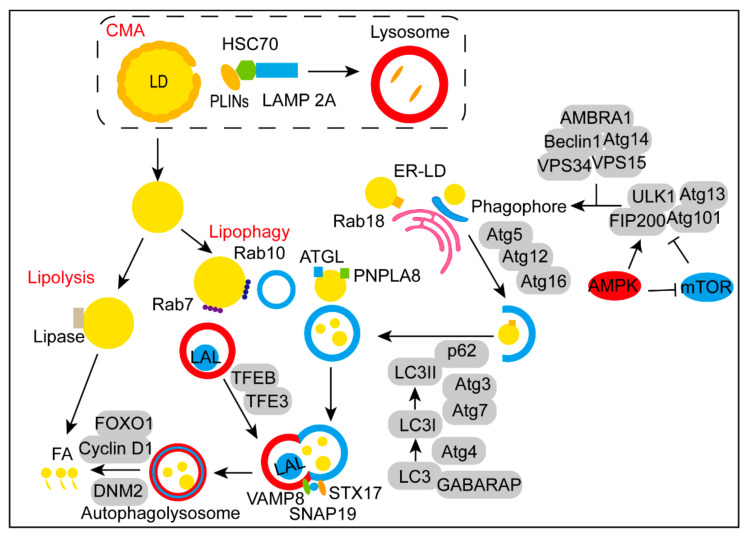
Steps of LDs degradation. After the degradation of PLIN2 and PLIN3 in lysosome via CMA mediated by HSC70, LDs are exposed for lipolysis and lipophagy. The activation of AMPK and mTOR recruits ULK1-Atg13-FIP200-Atg101 complex to trigger the initiation of autophagy. Next, the initiation complex composed of Beclin1, Atg14, VPS34, VPS15, and AMBRA1 is activated to form the origin of phagophore. Rab18 is essential in the spatiotemporal dynamic of ER-LD. During the elongation progress, the Atg5-Atg12-Atg16 complex promotes the autophagic vesicle formation. Simultaneously, Atg3, Atg4 and Atg7 are involved in the lipid-soluble form of LC3. Receptors such as p62 facilitate the localization of LC3-II on the autophagic membrane. On the surface of LDs, ATGL and PNPLA8 were identified as mammalian lipid receptors. Rab10 recruits LC3-positive phagophores to the circumference of LD, while Rab7 promotes the tethering and fusion of autophagosomes to lysosomes. TFEB and TFE3 are key regulators in autophagosome maturation and lysosomal biogenesis. Then, the VAMP8-SNAP29-STX17 complex mediates the form of autolysosome. Finally, LDs in the autolysosome degraded to FAs by LAL and regulated by FOXO1, Cyclin D1 and DNM2.

As a selective autophagy, the steps of lipophagy include initiation of autophagy, phagophore nucleation, expansion of phagophore, fusion of autolysosome and degradation of LDs, enjoying overlapping and special parts with autophagy (Figure 1).

#### 2.1.1. The Initiation of Autophagy via the Complex of ULK1-Atg13-FIP200-Atg101

Under tumor hypoxia and nutrient shortage circumstances, elevated levels of AMP/ATP and ADP/ATP ratios inhibit the mammalian target of rapamycin complex 1 (mTORC1) signal pathway by activating the energy sensor AMP-activated kinase (AMPK) pathway [20]. And the initiation of autophagy is triggered by the ULK1-Atg13-FIP200-Atg101 complex at the same time [21]. As the core component of the complex, the kinase activity of ULK1 is regulated by the phosphorylation level of Ser 317, Ser 467, Ser 574 and Ser757 [22]. Atg13, a lipid-binding protein, plays a mediating role and contains a HORMA domain [23]. FIP200 is the ortholog of Atg17 in mammals [24]. Atg101 binds to ATG13 through the HORMA domain to jointly maintain the stability of the complex [25]. Then the initiation complex composed of Beclin1, Atg14, VPS34, VPS15, and AMBRA1 is activated, leading to the accumulation of phosphatidylinositol 3,4,5-triphosphate (PI3P) at phagophore assembly site (PAS), which is the origin of phagophore [26]. Beclin1 is the core subunit of the complex [27], Atg14 determines the localization of the PI3K complex [28], VPS34 is mainly involved in the formation of endoplasmic reticulum (ER) membrane curvature [29], VPS15 plays a role in multiple membrane trafficking pathways [30], the major function of AMBRA1 is to positively regulate the lipid kinase activity [31].

#### 2.1.2. The Rab18-NRZ-SNARE Complex Is Essential in Establishing ER-LD Contact

Acting as gatekeepers of LDs, the degradation of LD surface proteins (PLINs) is a prerequisite for lipophagy to occur [7]. Exposure of the LD surface allows the phagophore nucleation step. Vesicular expansion and cisternal expansion are two possible mechanisms of phagophore nucleation, they rely on the delivery of lipid bilayers and the fusion of small compartments, respectively [32]. The dissociation of LDs from the ER is prior to phagophore nucleation. When it comes to the spatiotemporal dynamic of ER-LD, several proteins are involved. The Rab18-NRZ-SNARE complex has been proved essential in aggregating and establishing ER-LD contact to maintain LD formation. The deficiency of Rab18 led to a markedly decreased number of mature LDs and increased ER stress [33]. It has been found that DFCP1 was a Rab18 effector for LD localization and played a positive role in enhancing ER-LD contacts [34]. LDAF1-seipin complex is the core protein machinery in facilitating LD biogenesis and determining the location in the ER [35]. Meanwhile, the deficiency of ORP5 promoted the accumulation of triglycerides (TGs) in LDs by increasing the expression of PI(4)P, which is important in the transportation of phosphatidylserine [36].

#### 2.1.3. Rab10 Is Important in “Lipophagic Junction”

Many ATGs are critical in the elongation of phagophores. During the progress, the Atg5-Atg12-Atg16 complex promotes the autophagic vesicle formation [37]. Atg12 conjugates with Atg5 through a ubiquitin-like conjugating system and then bound to the N-terminal region of Atg16, which is paramount in recruiting the complex to autophagosomal membranes [38]. During the elongation of phagophore, another critical step is the binding of LC3/GABARAP family proteins, also called ATG8 family proteins to phosphatidylethanolamine (PE). Furthermore, Atg3, Atg4 and Atg7 are involved in the transition of LC3 from a soluble form (LC3-I) to a lipid-soluble form (LC3-II). Membrane curvature-reading domains in the N-terminus of Atg3 are required for the lipidation preferentially occurred in lipid-deficient membranes [39]. The Atg4 cysteine proteases are required for proteolytic activation and delipidation [40]. Atg7 an essential autophagy effector enzyme, activates the conjugation between either ATG5 and ATG12 or ATG8 family proteins and PE [41]. In line with it, overexpression of ATG7 reversed the accumulation of hepatic LDs [42]. Then, by interacting with receptors including p62, NBR1, NDP52 and OPTN, LC3-II is located in the lipid bilayer of the phagophore and delivers the modified target protein to phagophore [43]. Worth mentioning, Rab10 recruits LC3-positive phagophores to the circumference of LD surface via the form of Rab10-EHBP1-EHD2 complex, resulting a “lipophagic junction” [44].

#### 2.1.4. Rab7 Promotes “Lipophagic Synapses” in the Fusion of Autolysosome

Transcription factors TFEB and TFE3 are key regulators in autophagosome maturation and lysosomal biogenesis [45]. The local release of Ca2+ from lysosomes through mucolipin 1 (MCOLN1) activates calcium-dependent calcineurin, which binds and dephosphorylates TFEB, permitting its transcriptional activation [46]. The activated TFEB in nucleus next binds to CLEAR motifs, which comprise several promoters of lysosomal genes [47]. In addition, dephosphorylated TFE3 shuttles from cytoplasm rapidly to nucleus for combination of CLEAR elements, as a result, expanding the lysosomal compartment and facilitating autophagic flux [48]. Then the autophagosome docks with the lysosome with the help of dynein to form the autolysosome [49]. Importantly, Rab7, a fundamental component of LDs, promotes the progress termed “lipophagic synapses”, which induce the tethering and fusion of autophagosomes to lysosomes [50]. During the procedure, apart from VAMP8-SNAP29-STX17 complex [51], another complex YKT6-SNAP29-STX7 also mediates the fusion [52]. Molecules recruit to the progress include TECPR1 [53], EPG5 [54], PLEKHM1 [55] and GRASP55 [56], etc.

#### 2.1.5. Lysosomal Acid Lipase Degrades the Internal Lipids Stored in LDs

After the formation of autolysosome, lysosomal acid lipase (LAL), localized in the lysosome originally, attacks and degrades the internal cholesterol esters (CEs) and TGs stored in LDs [50]. The FAs produced by degradation are then released to the cytoplasm. Forkhead homeobox type protein O1 (FOXO1), a critical mediator of the cellular stress response, triggers lipophagy by up-regulation of LAL and then facilitates the lipid degradation cascade [11]. In addition, Cyclin D1 interferes with lipophagy by enhancing the accumulation of LDs or disrupting the degradation of LDs in the autolysosome [57]. Following the breakdown of LDs is the formation of new lysosomes by recycling autolysosomal membranes. During autophagic flux, the large GTPase DNM2 acted as a regulator of lipophagic turnover by promoting the scission of nascent tubules from previous autolysosome [58]. Under nutrient-limiting conditions, acting as precursors of protolysosomes, short tubular structures bud into cytoplasm to maintain efficient lipophagy operation [58].

#### 2.1.6. The Regulation of Lipophagy

On the one hand, many hormones are involved in the regulation of lipophagy. It is proved that β-adrenergic signaling enhances the lipophagy procedure in a Rab-7-dependent manner [59]. Stimulation of adipocytes with insulin or β-adrenergic receptor agonist isoproterenol benefits the supplementation of Rab18 to the surface of LDs [60]. Thyroid hormone 3 promotes lipophagy in a thyroid hormone receptor-dependent manner by activation of ANGPTL8 [61]. On the other hand, several transcriptional regulators and key enzymes are critical in the lipophagic progress. Under nutrient deprivation circumstances, the dephosphorylation of TFEB plays an essential role in enhancing lipophagy by the fasting transcriptional activator CREB and PPARα, while the activation of FXR could revise the procedure [62]. It is proved that SREBP-2 directly activates autophagy genes and increases LD turnover in the presence of cell-sterol depletion [63]. Lipophagic progress could be inhibited by abnormal accumulation of p62 and LDs as a result of superoxide dismutase 1 (SOD1) deficiency [64]. ATGL and PNPLA8 were identified as mammalian lipid receptors. Mechanistically, SIRT1 activity is required for ATGL-mediated induction of lipophagy [65] while PNPLA8 can interact with LC3 to induce lipophagy [66]. In addition, Muhammad et al. summarized varieties of natural compounds such as epigallocatechin-3-gallate (EGCG) [67], caffeine [68], bergamot [69] and resveratrol [70] could modulate lipophagy. It should be mentioned that researchs on the roles of dietary lipids level and stored lipid content in lipophagy are limited.

### 2.2. Associations between Lipophagy and Tumor Lipid Metabolism

**Figure 2:**
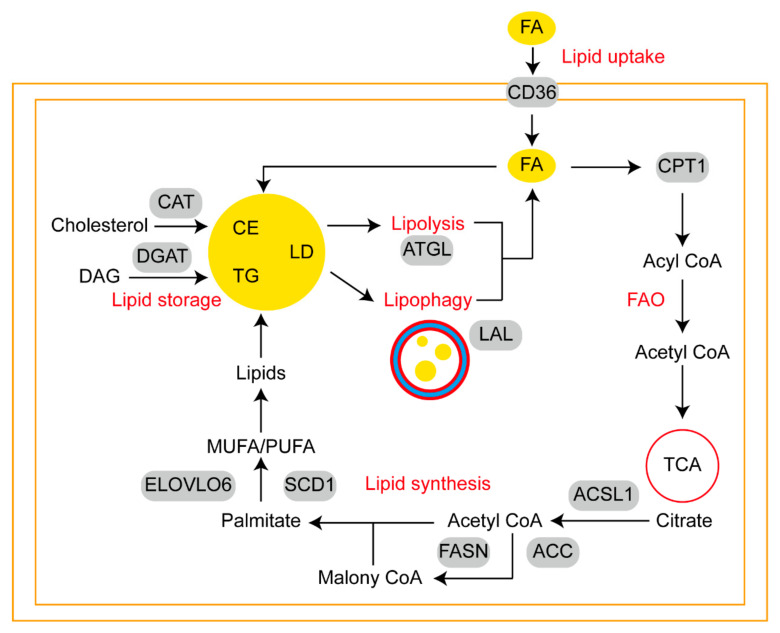
Schematic diagram of lipid metabolism reprogramming in metastatic tumor cells. Extracellular FAs could be transported across the membrane by CD36 and FABPs. The entry of FAs into mitochondria for FAO is controlled by CPT1. ACSL1 catalyzes the conversion of citrate to substrates for lipid synthesis. ACC and FASN are key enzymes in the biosynthesis of palmitate. Then, ELOVL6 and SCD1 facilitate the conversion of monounsaturated fatty acid (MUFA) and polyunsaturated fatty acid (PUFA). Excess lipids can be stored in the forms of LD, which cores are CE and TG. Under stress conditions, LDs could be degraded by lipolysis and lipophagy to generate energy.

Cross-talk between adipocytes and cancer cells in the tumor microenvironment (TME) can create a metabolic symbiosis driving cancer metastasis. Lipophagy can occur in both adipocytes and tumor cells. Lipophagy may modulate differentiation process of adipocytes from adipocyte precursor cells. Research carried out by Hahm et al. showed that the expression of LC3-II, AMPK activity and formation of acidic vesicles appeared during the normal differentiation of mouse 3T3-L1 preadipocytes [71]. The inhibition of lipophagy resulted in the inhibition of the adipogenic differentiation of embryonic fibroblasts by preventing LDs aggregation [72]. Adipose tissue can be fertile ground for metastatic tumors, for example, melanoma actively absorbs lipids and is more likely to metastasize to adipocyte-rich tissue [73]. In similar, ovarian cancer has a clear predilection for metastasis to omentum, a fatty tissue characterized by immune structures called milky spots. Milky spots and adipocytes are responsible for the localization of disseminated cancer cells and subsequent growth respectively [74]. Lipids absorption by tumor cells facilitate remodeling of lipid metabolism (Figure 2), which includes five parts: (1) lipid uptake; (2) fatty acid oxidation (FAO); (3) lipid synthesis; (4) lipid storage; (5) lipolysis and lipophagy.

#### 2.2.1. Sources of Fatty Acids Available to Tumor Cells

Known as energy sources and membrane constituents, FAs are required to meet the metabolic demand of cancer cells or to adapt to reduced serum-derived lipid availability in TME [75]. A variety of FAs derived from dietary routes and endogenous. Nutrients consumed by tumor cells may affect cancer progression, dietary polyunsaturated fatty acid (PUFA) as a selective adjuvant antitumor modality may efficiently induce ferroptosis in acidic cancer cells [76]. During feeding, gut microbes produce short-chain fatty acids (SCFAs), which promote prostate cancer growth via IGF1 signaling [77]. Metastasis-associated tumor stromal cells, such as adipocytes, fibroblasts, and macrophages, are frequently sources of FAs. During adipocyte lipolysis, FAs provide breast cancer cells with metabolic substrates [78]. FAs are transferred from cancer-associated fibroblasts to cancer cells through vesicles, which promote tumor growth [79]. Macrophages are a rich source of FAs, in addition, increased levels of FAO in macrophages can effectively promote M2 polarization [80]. To some extent, cancer cell growth relies on FAs supplied through the bloodstream [81].

#### 2.2.2. CD36 and Fatty Acid-Networks Binding Proteins Participate in Lipid Uptake

Tumor cells can support growth by ingesting FAs obtained from extracellular lipolysis. In normal physiological state, as essential membrane glycoprotein, CD36 mediates lipid uptake, immunological recognition, inflammation, molecular adhesion, and apoptosis in multiple cell types [82]. CD36, a scavenger receptor, is highly expressed in tumor cells at metastatic sites but low in the carcinoma in situ [83]. The large extracellular region of CD36 is used for the binding of FAs and oxidized LDL by forming hydrophobic cavities [84]. CD36 is transported across organelles and cell membranes by vesicles, regulating FA uptake and metabolic energy balance. It has been shown that uptake of PA by CD36 leads to AKT phosphorylation, which is positively correlates with gastric cancer metastasis [85]. The report by Ladanyi et al. suggested that ovarian cancer (OvCa) cells co-cultured with primary human omental adipocytes express high levels of CD36 in the plasma membrane, thereby facilitating exogenous FA uptake and OvCa metastasis [86]. In similar to the function of CD36, fatty acid-networks binding proteins (FABP) are lipid chaperones assisting in the trafficking of fatty acids [87]. Decreased expression of FABP2 and FABP3 was observed when adding lipids into MDA-MB-231 cells pretreated with fibroblast media. Concomitant with this was an increase in FAs uptake and a decline of fatty acid synthase (FASN) activity [88]. Targeting FABP4 can inhibit the ability of ovarian cancer cells to adapt and colonize in lipid-rich TME efficiently [89,90]. It is worth noting that activation of the FABP12/PPARγ pathway in prostate cancer cells facilitates metastatic transformation via energy derived from lipids [91].

#### 2.2.3. Carnitine Palmityl Acyltransferase 1 Is the Key Enzyme in FAO

The ingested FAs are then utilized by FAO, also called β-oxidation, which mainly occurs in mitochondria. The progress requires long-chain acyl-coenzyme A synthase (ACSL) to activating acyl CoA, which is then catalyzed by carnitine palmityl acyltransferase 1 (CPT1) from cytoplasm to mitochondria. Each ACSL isoform is responsible for channeling long-chain FAs to a different metabolic fate. Its deregulation has also been observed frequently in variable cancers [92]. A miR-377-3p-CPT1 axis has been implicated in the metastasis of hepatocellular carcinoma through mediating FAO [93]. By repeating multiple rounds of enzymatic dehydrogenation, hydration, dehydrogenation and thiolysis, acetyl-CoA, NADH and FADH2 can enter the TCA cycle or maintain cellular homeostasis. YAP, a transcriptional coactivator, is selectively activated in lymph node-metastatic tumors, resulting in increased expression of genes involved in FAO signaling [94].

#### 2.2.4. FASN Plays Important Roles in Lipid Synthesis

As ATP citrate lyase (ACLY) converts citrate to oxaloacetate, cytosolic acetyl-CoA is generated, which serves as a major substrate for lipid synthesis. The IKKβ-USP30-ACLY axis controls lipogenesis and tumorigenesis [95]. First committed step, the synthesis of malonyl-CoA is catalyzed by acetyl-CoA carboxylase (ACC). Lipogenesis and hepatocellular carcinoma are suppressed when ACC is inhibited by phosphorylation or ND-654 [96]. Multiple polymerizations of acetyl-CoA and malonyl-CoA under the catalytic action of FASN create saturated fatty acids. In the progression of breast cancer, CircWHSC1regulates the FASN/AMPK/mTOR axis via the sponge of miR-195-5p [97]. Palmitate, the product of FASN-mediated de novo lipogenesis, can either be elongated by very long chain fatty acids protein 6 (ELOVL6) or desaturated by stearoyl-CoA desaturases (SCDs). ELOVL6 regulates bortezomib resistance in multiple myeloma [98]. SCD1 has been identified as a mechanoresponsive enzyme in response to matrix stiffness and lipid metabolism and its overexpression is a prognostic marker associated with poor outcomes in HCC patients [99].

#### 2.2.5. LD Is a Major Form of Lipid Storage

An excess of FAs can be harmful to cells due to lipotoxicity, so cells convert FAs into TGs and CEs for storage in LDs. The final step of TG synthesis is catalyzed by diacylglycerol acyltransferase (DGAT) while cholesterol acyltransferase (CAT) is the key enzyme in the form of CEs. By the way, some extra sphingolipids and cholesteryl esters can form lipid rafts in the plasma membrane that play a role in signal transduction [100]. As a result of the anabolic reaction, neutral lipids are continuously deposited in a lens-like microdomain between the ER bilayer membranes, the outer lobe of which buds to form a distinct spherical organelle, the LDs. Then, the initial LDs could be converted to expanding LDs and giant LDs. In response to an increase in cellular FAs levels, LD biogenesis is stimulated. Of note, acidic microenvironment caused by tumors favors the synthesis and release of LDs [101,102]. LDs vary in size and number in different cell types, revealing the capacity of managing lipid storage [103]. Moreover, LDs are thought to mediate lipid transport by directly interacting with most cellular organelles [104].

#### 2.2.6. ATGL Predicts a Linear Relationship between Lipolysis and Lipophagy

Malnutrition and stress conditions mediate LDs degradation via lipolysis and lipophagy. In colon cancer cells, overexpression of key lipase ATGL is observed in obesity-promoted colonic tumorigenesis via lipid metabolic reprograming [105]. Meanwhile, Elevated DECR1 directly activates HSL to enhance lipolysis in Hela cells [106]. It has been demonstrated that, under glucose deprivation, choline kinase (CHK) promotes lipolysis, FAO and brain tumor growth by phosphorylating PLIN2/PLIN3 on the surface of LDs [107]. In a recent study, Satyanarayan and colleagues found that there was a linear relationship between lipase ATGL and lipophagy. By increasing the interaction between LC3 and lysosomes with LD, ATGL increases lipophagy and positively regulates autophagy in the liver [65]. Of note, studies indicated that lipolysis preferentially targeted larger-sized LDs in hepatocytes, while the resulting small LDs (<1 μm^2^) are more suitable for lipophagy [108]. Whether a similar phenomenon exists in tumor lipid metabolism remains to be further demonstrated.

## 3. Evidence of Lipophagy in Cancer Metastasis

According to integrative analysis of multiple omics data, it is revealed that lipid metabolism disturbances caused by lipophagy may confer pro-metastatic traits and directly impact the metastatic dissemination process [109]. The metastases are formed after the completion of a complex series of cellular biological events named as invasion-metastasis cascade. As the process unfolds, cells break free from the primary tumor [110]. The tumor cells then penetrate the blood vessels via angiogenesis and proteolysis [111]. Cancer cells are subsequently found in the circulation either as single cells or as emboli containing platelets or leukocytes to protect them from shear forces, immune attack, and anoikic [112]. Then, tumor cells arrest at distant organs and extravasate into the parenchyma. Following extravasation, the vast majority of cells die while the rest remain dormant or grow into a micrometastasis [113]. As a result of adaptation and remodeling of the microenvironment, cancer cells form a macrometastatic lesion [3]. This whole cascade is orchestrated by a cohort of molecular pathways such as epithelial–mesenchymal transition (EMT), together with genes related to oxidative stress, viral replication and signal transduction. Given the limited researchs are available on the role of lipophagy in cancer metastasis, we will clarify the role of lipophagy in several of the above-mentioned biological process related to cancer metastasis in this section.

### 3.1. The Interaction between Lipophagy and EMT

EMT is a cellular pathway that represents a salient property of primary tumor development and metastasis, in which cells lose their epithelial properties (characterized by the loss of membranous E-Cadherin) and acquire features of mesenchymal cells (featured by increased N-cadherin, vimentin expression along with migratory capability) [114]. Higher levels of MUFA together with enhanced de novo fatty acid synthesis is the unique signature of epithelial cells. By contrast, reduced lipogenesis, higher PUFA level and increased TGs synthesis and LDs formation are observed in mesenchymal cells [115]. There is evidence that the loss of C/EBPδ, a critical lipid metabolic regulator can induce significant oscillations in EMT gene networks, leading to cancer metastasis via enhanced oxLDL uptake [116]. In hepatocellular carcinoma, lipophagy has been proved to participate in fluid shear stress (a pathological factor in cancer metastasis)-induced EMT and cell migration [117]. It is revealed that inhibition of 14,15-epoxyeicosatrienoic acid, an important lipid signaling molecule could serve as a novel strategy to reverse EMT in breast cancer cells [118]. Exploring the role of the lipophagy portion of lipid signaling in EMT is integral to elucidating the mechanisms of cancer metastasis.

### 3.2. Oxidative Stress and Lipophagy

Oxidative stress refers to a state in which the oxidative and anti-oxidative effects are unbalanced. The accumulation of reactive oxygen species (ROS) is the main cause of it [119]. To optimize ROS-driven proliferation, cancer cells enhance the capacity of anti-oxidation while avoiding ROS thresholds that are necessary for senescence, apoptosis, or ferroptosis [120]. Ferroptosis is a type of nonapoptotic cell death caused by lipid peroxidation. Recent studies revealed that lipophagy promoted RSL3-induced lipid peroxidation and subsequent ferroptosis in hepatocytes, which can be prevented either by enhancing TPD52-dependent lipid storage or blocking ATG5- and RAB7A-dependent lipid degradation [121]. Lipophagy mediated by PGRMC1 is required for ferroptosis in paclitaxel-tolerant persister cancer cells [18]. Interestingly, a recent study revealed a unique relationship between ATG14 and lipophagy in HeLa cells. In response to ATG14 overexpression, lipophagy and intracellular FAs accumulation occur, which lead to oxidative stress and apoptosis, while inhibition of ATG14 maintains cell survival [122]. In addition, it was previously found that oxidative and ER stress pathways played crucial regulatory roles in high glucose-induced the activation of lipophagy and lipid metabolism via enhancing the DNA binding capacity of carbohydrate response element-binding protein at PPARγ promoter region, which in turn induced transcriptional activation of the key genes related to lipogenesis and lipophagy [123]. Similar pathogenic mechanisms have not been investigated in tumor patients with obesity, which is a risk factor in certain cancers.

### 3.3. Lipophagy in Viral Replication

Lipophagy is a selective autophagic process that not only facilitates the clearance of lipid aggregates, but also provides energy for the replication of multiple pathogenic microorganisms. In the latest study, inflammatory mediators released by LDs and their surface antiviral proteins form a line of defense during microbial infection, thereby acting as a molecular switch in innate immunity [124]. Hepatitis virus infection often exacerbates the vascular metastasis of liver cancer. In liver cancer cells, lipophagy leads to a significant decrease in LD volume and TG, which contributes to the replication of hepatitis C virus (HCV) [125]. In a recent study, LDs-associated ABHD5 was essential for the assembly and release of HCV, the underlying mechanisms by which it mobilized LDs-associated lipids metabolism is possibly via the regulation of ATGL activity [126]. Additionally, inhibition of autophagy increases cellular oxidation, which prevents the lipophagy of virus [127]. Furthermore, the small GTPase Rab11a has been identified to function as a proviral host factor in stimulating viral replication [128], and there is evidence that the silence of Rab11b leads to the accumulation of LC3-II [129], whether Rab11a is essential in viral replication through lipophagy remains to be further explored.

### 3.4. Lipophagy in Signal Transduction

As a result of lipophagy, excessive FAs are produced in lysosomes. Researchers recently discovered that lipid-filled lysosomes fuse with plasma membranes for extracellular export, altering systemic lipid homeostasis and intercellular communication [130]. P53 is a tumor-suppressor that plays a direct role in the regulation of autophagy and FAO-related genes, as well as promoting lipid degradation afterwards [131]. LC3-II and perilipins, both of which are necessary for lipophagy, can be downregulated by inhibition of p53, which is required for the induction of lipophagy in hepatoma cells. Thereby, p53 exerts a unique tumor-suppressor role through lipophagy [132]. It has been documented that LAL deficiency can increase the risk of tumorigenesis and metastasis via mTOR signaling [133]. Moreover, by activating PI3K/AKT/mTOR, Apolipoprotein C-II promotes gastric cancer peritoneal metastatic spread [134]. By altering lipid metabolism, cancer stem cells (CSCs) can both satisfy their energy demands and produce biomass, as well as stimulate a number of key oncogenic signaling pathways, such as Wnt/β-catenin and Hippo/YAP signaling [135]. However, whether these signaling pathways affect malignant metastasis via lipophagy needs to be further studied.

## 4. Potential Treatment Strategies through Targeting Lipophagy

With the increasing understanding of the mechanism of lipophagy, scholars try to find ways to effectively control metastasis by inhibiting the lipophagy pathway. There have been recent proposals that LDs can serve as functional markers of CSCs, whose biogenesis is a multistep process involving the enzyme diacylglycerol acyltransferase 2 (DGAT2). Pretreatment with PF-06424439, an inhibitor of DGAT2, in conjunction with X-ray exposure can enhance the radiosensitivity of breast cancer cells and improve the effectiveness of radiotherapy [136]. When used alone and in combination with carboplatin, AACOC3 effectively inhibited tumor growth and LD biogenesis in ovarian cancer [137]. Taking metformin works in reducing adipocyte secretion of MCP-1 and prevents ovarian cancer metastasis by inhibiting MCP-1/CCR-2 axis [138]. By activating a variety of pathways involved in apoptosis and lipophagy, simvastatin can kill radioresistant breast cancer cells, as well as inhibit their migration abilities and vimentin expression [139]. An inhibitor of PFKFB3 called PFK-15 promotes lipophagy in gynecologic cancers and is also chemosensitive [140]. Resveratrol, a tumor-suppressor, reverses lysophosphatidic acid induced cell migration and platinum resistance via rescuing hedgehog-mediated autophagy in ovarian cancer therapy [141]. Hydroxychloroquine (HCQ) and chloroquine are inhibitors of autophagy used in the cancer clinic. Goldberg and colleagues have successfully demonstrated the safety of HCQ with and without erlotinib in the treatment of non-small-cell lung cancer patients [142]. Over expression of CCAAT enhancer-binding protein a in hepatocellular carcinoma weakens tumorigenesis by increasing lipophagy, which can be abrogated by chloroquine treatment [143]. Celastrol, a triterpene derived from the traditional Chinese medicine, has been identified as a lipophagy-based anticancer therapeutic approach in ccRCC [144]. Additional studies indicated that melatonin promoted “tumor slimming” and suppressed ccRCC development via PGC1A/UCP1-mediated autophagy and lipid browning [145]. Aside from developing new targeted drugs, researchs on new applications of old drugs are also urgently needed.

## 5. Conclusions

The above summarized the crosstalk among lipophagy, tumor lipid metabolism and cancer metastasis. A better understanding of the crosstalk mechanism will lead to new and exciting therapeutic possibilities for cancer elimination. Despite the fact that the related mechanisms of lipophagy have been illustrated, as well as some proteins or genes that are key participants in lipophagy have been validated, the overall landscape of lipophagy requires further research. The researchs on the roles of lipophagy in tumor cells and tumor stromal cells including tumor-associated adipocytes, tumor-associated fibroblasts and tumor-associated macrophages are also warranted. Unfortunately, the mechanism of lipophagy related to tumor angiogenesis and immune escape is still lacking, which are closely related to the metastatic potential of tumors. With the development of new sequencing technologies and probe labels, more pivotal factors involving lipophagy and cancer metastasis will be formulated, and new personalized clinical treatment options will be developed, based on the sufficient fundamental research.

## Abbreviations

LD: Lipid droplet; FA, fatty acid; PLIN, lipid droplets surface protein; ER, endoplasmic reticulum; TG, triglycerides; LAL, lysosomal acid lipase; CE, cholesterol esters; TME, tumor microenvironment; FAO, fatty acid oxidation; MUFA, monounsaturated fatty acid; PUFA, polyunsaturated fatty acid; FABP, fatty acid-networks binding proteins; FASN, fatty acid synthase; ACSL, long-chain acyl-coenzyme A synthase; CPT1, carnitine palmityl acyltransferase 1; ACLY, ATP citrate lyase; ACC, acetyl-CoA carboxylase; ELOVL6, very long chain fatty acids protein 6; SCD, stearoyl-CoA desaturase; DGAT, diacylglycerol acyltransferase; CAT, cholesterol acyltransferase; EMT, epithelial–mesenchymal transition; ROS, reactive oxygen species.
